# Cortical Representation and Excitability Increases for a Thenar Muscle Mediate Improvement in Short-Term Cellular Phone Text Messaging Ability

**DOI:** 10.3390/brainsci11030406

**Published:** 2021-03-23

**Authors:** Anthony W. Meek, Joselyn Perez, Brach Poston, Zachary A. Riley

**Affiliations:** 1 School of Health and Human Sciences, Indiana University-Purdue University, Indianapolis, IN 47405, USA; antmeek@iupui.edu (A.W.M.); joselyn.perez@uconn.ed (J.P.); 2 Department of Kinesiology and Nutrition Sciences, University of Nevada-Las Vegas, Las Vegas, NV 89154, USA; brach.poston@unlv.edu

**Keywords:** transcranial magnetic stimulation, cortical representations, text messages

## Abstract

Cortical representations expand during skilled motor learning. We studied a unique model of motor learning with cellular phone texting, where the thumbs are used exclusively to interact with the device and the prominence of use can be seen where 3200 text messages are exchanged a month in the 18–24 age demographic. The purpose of the present study was to examine the motor cortex representation and input–output (IO) recruitment curves of the abductor pollicis brevis (APB) muscle of the thumb and the ADM muscle with transcranial magnetic stimulation (TMS), relative to individuals’ texting abilities and short-term texting practice. Eighteen individuals performed a functional texting task (FTT) where we scored their texting speed and accuracy. TMS was then used to examine the cortical volumes and areas of activity in the two muscles and IO curves were constructed to measure excitability. Subjects also performed a 10-min practice texting task, after which we repeated the cortical measures. There were no associations between the cortical measures and the FTT scores before practice. However, after practice the APB cortical map expanded and excitability increased, whereas the ADM map constricted. The increase in the active cortical areas in APB correlated with the improvement in the FTT score. Based on the homogenous group of subjects that were already good at texting, we conclude that the cortical representations and excitability for the thumb muscle were already enlarged and more receptive to changes with short-term practice, as noted by the increase in FTT performance after 10-min of practice.

## 1. Introduction

The topography, or distribution of a muscle’s cortical neurons in primary motor cortex (M1), is important in motor skill learning where there is an orderly relationship between the neurons and the external input/output [1]. There is re-organization with motor learning that results in a change in the topographic motor ‘map’ or the distribution and connectivity of M1 neurons where representative areas expand or contract [2]. This has been shown directly in rats trained to perform reaching movements where the intrinsic horizontal synaptic connections within layers II/III of M1 were strengthened [3]. The evidence of LTP-like plasticity in the forelimb region of M1 is similar to what is observed in humans performing repetitive motor tasks (piano exercises) where there is an increase in the cortical representation when mapped with transcranial magnetic stimulation (TMS) [4].

Motor skill learning and plasticity in M1 have to be considered relative to the practice duration as it has been established that there are two-phases to motor skill learning, each driven by individual physiological adaptations [5,6,7]. The initial phase of adaptation can occur in as little as 5–10 min of practice, though it is more commonly observed with a longer durations [8], and can be due to the disinhibition of intracortical inhibitory circuits [6] or the unmasking of existing subthreshold excitatory connections [2]. Alternatively, the later phase of motor skill adaptation requires repeated practice over several days or more and can be due to intracortical synapses being maximally strengthened [9], a greater transfer of information between M1—basal ganglia—cerebellum [4], and/or synaptogenesis [10]. Depending on the phase of motor skill learning, there are different mechanisms in place for the nervous system to adapt and consolidate practice of a task to improve performance.

A novel way of studying motor skill learning and associated M1 plasticity is with cellular phone text messaging, where in the United States alone over 94% of adults ages 18–24 own smartphones; this demographic averages more than 3200 text messages exchanged a month (pewinternet.org). Given the design and orientation of the standard smartphone, all typing is accomplished by moving the thumb(s) repeatedly over the screen surface. This creates a unique model where the thumb is used excessively in daily living, presumably enlarging the cortical representation in muscles controlling that digit, similar to what has been shown in the first dorsal interosseus muscle (controlling the index finger) in experienced braille readers [11]. However, along with enlargement of the representation in first dorsal interosseus, Pascual-Leone et al. found a reduction in the size of the representation of the abductor digiti minimi (ADM) muscle, which is not typically used in braille reading. These findings suggest that highly specialized sensorimotor skills cause cortical reorganization that may be necessary for task proficiency, and the reorganization can lead to a use-dependent plasticity in the intracortical networks.

The purpose of the present study was to examine the motor cortex representation and input–output (IO) recruitment curves of the abductor pollicis brevis (APB) muscle of the thumb and the ADM muscle with transcranial magnetic stimulation (TMS), relative to individuals’ texting abilities on a cellular phone. Towards this purpose, a functional texting task (FTT) was devised to study individual texting performance. A functional keyboard task (FKT) was also developed as a way to study general typing abilities and what may be a confounding factor in focal development of the APB representation. A practice FTT was then performed for 10-min and the mapping and input–output curves were repeated to examine short-term plasticity in the cortex of the subjects. The hypothesis was that the motor map of the thumb would be more extensive, and there would be more stimulus-intensity dependent recruitment of corticospinal projections to the thumb [12], in those individuals with accomplished texting ability. Additionally, we expected that these cortical changes would be more prominent following the short-term practice of the FTT. Finally, we expected these changes in the APB to be accompanied by decreases in map representation size and excitability for the ADM, though less so for individuals with higher FKT scores due to the distribution of intracortical networks being divided among all digits involved in typing.

## 2. Materials and Methods

### 2.1. Participants

Eighteen healthy individuals participated in the study (24.1 ± 4.0 years old; range: 20–34). Subjects self-reported no history of psychiatric disease, neurological problems, history of seizures or epilepsy, recent history of injury or disease involving the upper dominant extremity, pacemaker or other metal implants in the upper body, and negative pregnancy test (for women of childbearing potential). Subjects completed an Edinburgh Handedness Inventory [13], with 16/18 being identified as right-handed. The Indiana University Human Subjects IRB Committee approved the protocol (#1703811075) and all subjects provided informed consent before participating.

### 2.2. Experimental Setup

**Figure 1:**
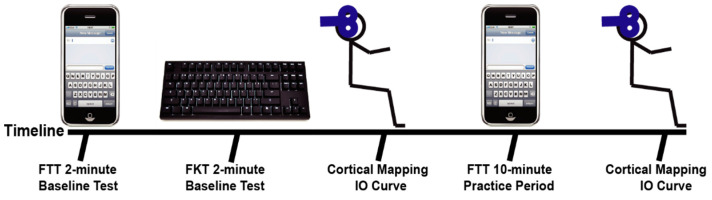
Experimental timeline for the functional texting task and functional keyboard task baseline test (FTT and FKT), baseline cortical mapping and IO curves, practice period, and the final cortical mapping and IO curves.

Subjects were given instructions on the FTT, the FKT, and were briefed on the TMS procedures. Subjects performed a timed FTT where they completed as many 45-character long, random phrases as possible in 2-min on a cellular phone and were scored for their pre-test (see Figure 1). Subjects then performed an FKT in which they completed an additional 45-character long, random phrases on a traditional computer keyboard as possible. The subjects were next seated comfortably in an upright position with the right arm resting on a padded table and placed in 60° shoulder abduction and 90° elbow flexion. Transcranial magnetic stimulation (TMS) was used to examine the cortical representations and IO recruitment curves for the APB and ADM muscles. When the initial cortical stimulation was complete, subjects performed a 10-min practice FTT. The 10-min practice FTT was self-paced, but subjects were again presented with random 45-character long phrases and were encouraged to complete as many as possible in the 10-min period. After the 10-min practice FTT, the TMS procedures were repeated to examine any short-term changes that may have occurred with practice of the FTT. Finally, the subjects completed a final 2-min FTT to determine performance effects of the 10-min of texting practice. The post-test FTT was performed at least 10 min after the practice FTT, so the subject had sufficient time to recover.

### 2.3. Functional Texting Task and Functional Keyboarding Task

Subjects performed the FTT using their own personal, modern, touch-screen cellular phone with a full keyboard, and the phone oriented vertically (Android or iPhone, ≥4 in screen). The ‘auto-correct’ feature was turned off prior to using the phone for the task. A custom written program in MATLAB (Mathworks, Natick, MA, USA) would display the random 45-character phrases and the subjects were asked to enter those phrases as accurately and quickly as possible. After each phrase, they would click the ‘send’ button and then move on to the next phrase. Subjects were informed to ignore capitalization and not to delete or go back when they made an error. Each subject was given an absolute texting score based on the total number of correct characters produced. Relative accuracy was also calculated by dividing the number of correct characters entered by the total number of characters entered. The FKT was performed with the subjects using a desktop computer keyboard on a blank Microsoft Word document. Random 45-character sentences were again displayed for the subjects, and at the end of each sentence the subjects would hit enter and proceed to the next sentence. The FKT was scored in the same way as the texting task.

### 2.4. Electromyographic Recordings

Electromyographic (EMG) activity resulting from the TMS procedures was collected from the APB and ADM muscles of the dominant hand. A single differential detection electrode was placed over the muscle belly of each muscle. The skin overlying each muscle was cleaned and lightly abraded prior to affixing the electrode. A single common ground electrode was placed over the acromion process of the dominant side of the body. The signals were amplified and conditioned (Bagnoli EMG System, Delsys Inc. MA, USA) with high- and low-pass cut-off frequencies of 20 Hz and 1000 Hz, respectively, before being stored at a final gain of 2000x with Spike2 software (CED, Cambridge, UK) for subsequent analysis.

### 2.5. TMS Procedures

Single transcranial magnetic stimuli were delivered using a standard Magstim 200^2^ stimulator (Magstim Company LTD, UK) with a 70 mm figure-of-eight shaped coil. The coil handle was pointing backwards and laterally ~45° to the interhemispheric line while the subject wore a tight-fitting nylon cap. Stimulation was delivered over the left or right hemisphere, contralateral to the dominant hand. With the subject relaxed, supra-threshold stimulation was used to determine the optimal position for stimulation of the APB cortical representation. After determining the optimal position, the resting motor threshold (RTh) was determined with step-wise increases in stimulator output. Threshold was determined when motor-evoked potential (MEP) responses were greater than 50 μV in 5 out of 10 consecutive stimuli. The site was marked on the cap and a 5 × 6 cm grid with 1 cm grid spacing was placed on the cap with the center of the grid matched with the marked optimal stimulation site. The grid size was chosen to completely encompass the APB and ADM representations based on the size of the motor areas and the amount of overlap between the two areas as reported by Wilson et al. [14]. TMS intensity during the mapping protocol was set at 120% RTh based on the response at the optimal stimulation site for APB. Three stimuli were delivered at each grid site with an inter-stimulus interval of at least 2 s, and the average response was recorded. Three stimuli are considered sufficient to develop a reliable cortical map [15]. The mapping procedures were performed at least 5 min after the initial FTT and FKT, and at least 2 min after the practice FTT.

### 2.6. Data Analysis

The maximum peak–peak amplitude was determined from the average of the 3 MEP responses at each grid point stimulation site where the MEP was evident. Map volume was then determined as the sum of the responses at all of the active sites [16,17]. The map area was determined as the number of active sites in the grid. In addition, the center-of-gravity (CoG), or the position of the motor map that demonstrated the highest amplitude response to stimulation [18], was examined before and after the practice FTT.

The cortical mapping site that had the highest combined response between the pre-test and post-test stimulation in APB was examined further. For this stimulation site, we also compared the responses at 120% RTh before and after practice for both the APB and ADM muscles.

### 2.7. Statistical Analysis

Paired *t*-tests were used to compare the FTT and FKT scores and accuracy. T-tests were also used to compare the FTT scores, accuracy, and the cortical stimulation variables before and after practice. Pearson’s r correlation was used to determine the similarity between the texting variables (score and accuracy) and the mapping variables (normalized map volume and number of active sites) and 120% RTh responses before the 10-min practice. Finally, correlations were also used to examine the percent change from pre–post practice in the FTT with the percent change in the cortical variables. Significance level was set at *p* < 0.05. All statistical tests were performed with the Statistics Toolbox in MATLAB.

## 3. Results

**Figure 2:**
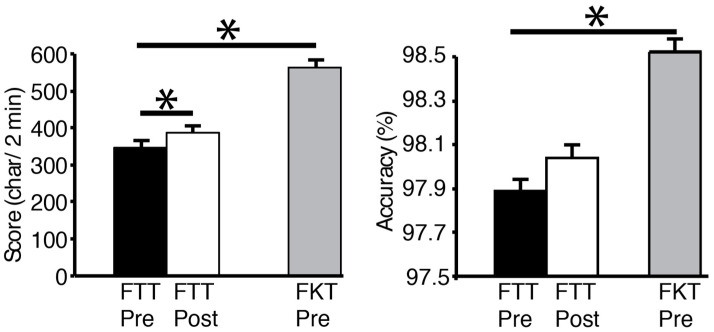
Score and accuracy of the FTT for pre and post practice, in comparison to the pre-test of the FKT. Scores significantly increased in the FTT from pre to post, but were significantly lower than the FKT during the pre-testing period. Subjects were more accurate with the FKT than the pre-test FTT. However, the FTT accuracy did not change after practice. * denotes significance (*p <* 0.05)**.**

All FTT and FTK scores showed a normal distribution. Subjects’ scores averaged 346.6 ± 69.5 characters correctly typed in the FTT pre-test. The number of characters in the FKT (563.6 ± 89.4) was significantly higher than the FTT (*p* < 0.001), and the accuracy was better as well (98.5 ± 2.3%, vs. 97.9 ± 2.3%, *p* = 0.04, Figure 2). After the 10-min practice FTT, the texting score went up to 386.9 ± 71.2 characters, which was significantly different than the pre-test FTT (*p* = 0.047). Texting accuracy before and after the practice was not different (*p* = 0.38). Interestingly, as an aside, there was a negative correlation between the pre-test FTT and age (*r =* −0.74, *p* = 0.005).

There were no correlations between any of the cortical mapping variables (volume, area) or the 120% RTh MEPs for the APB and the pre-test score (initial 2-min test) on the FTT. Similarly, there were no correlations with the cortical testing procedures for either APB or ADM and the FKT.

**Figure 3:**
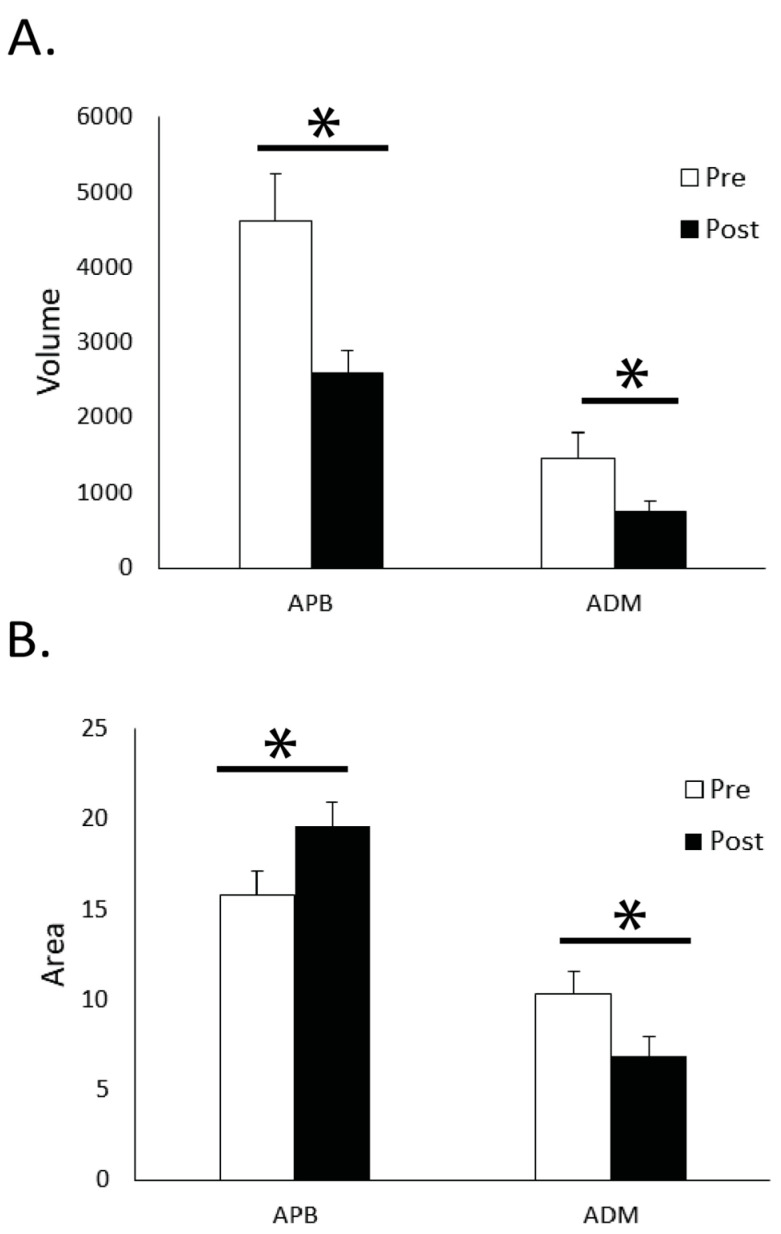
(**A**) Cortical volume in the abductor pollicis brevis and abductor digiti minimi muscles from pre–post texting practice. Volume significantly decreased for both APB and ADM muscles. (**B**) Cortical area significantly increased in the APB, and significantly decreased in the ADM from pre–post texting practice. * denotes significance (*p <* 0.05)**.**

**Figure 4:**
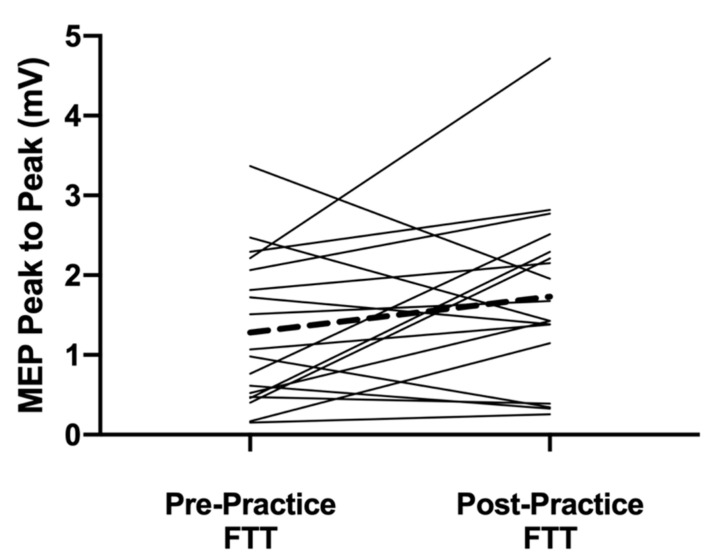
Motor-evoked potential size at an intensity 120% of resting motor threshold before and after the practice FTT for each individual subject. The dashed line denotes the mean change for the group. There was a significant increase in MEP size after practice (*p* = 0.04)**.**

The main results from the cortical mapping procedures were observed after the 10-min FTT practice session. The cortical volume for APB significantly decreased (*p* = 0.001) after the practice period, while the area increased from 15.8 ± 5.7 active sites before practice to 19.6 ± 5.5 after practice (*p* = 0.004, Figure 3). A difference was also observed with an increase for the APB in the 120% RTh MEPs after practice (*p* = 0.042, Figure 4). With the ADM, the cortical volume decreased after practice (*p* = 0.002), but the area also decreased (10.3 ± 5.7 to 6.8 ± 4.6; *p* < 0.001). The 120% RTh MEPs for ADM did not change after practice.

**Figure 5:**
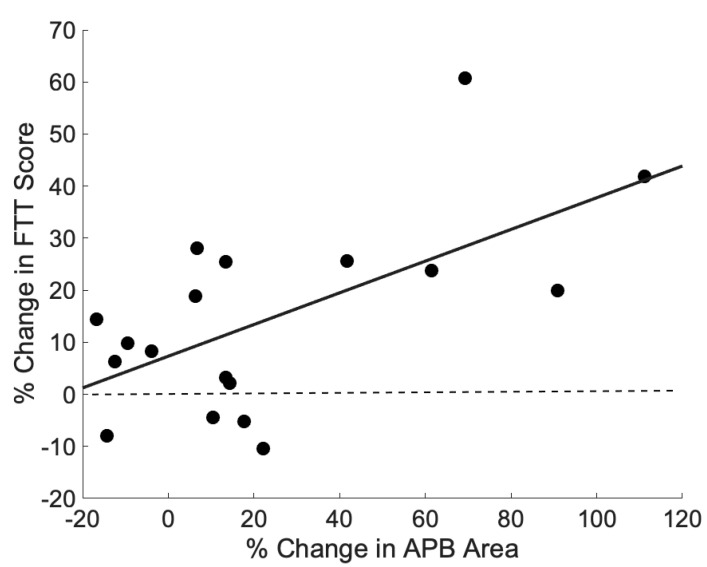
Plot of the percent change in cortical map area versus the percent change in FTT score for all 18 subjects (*r =* 0.61, *p* = 0.007). A simple linear regression line was fit to the data.

There was a significant correlation between the percent change in APB area and the percent change in pre–post FTT scores (*r =* 0.61, *p* = 0.007, see Figure 5). None of the other changes in APB cortical measures (volume, 120% RTh) correlated with the percent change in FTT. Similarly, the change in ADM cortical measures did not correlate with the percent change in FTT.

## 4. Discussion

The main finding of the present study is that individuals are proficient at cellular phone text messaging, though significantly slower than traditional keyboard typing. As keyboard typing still involves the use of all digits of both hands, this may explain why there were no associations present between cellular phone texting ability and the cortical variables for the thumb muscle that we measured before practice. However, it was shown that short-term plasticity in the APB representation occurred with practice of the texting task where the map volume decreased, but the area (number of stimulation sites) and MEP size at 120% RTh both increased while the subjects improved at the FTT. Along with this result, the increases in the FTT scores were correlated with the increases in the APB area. Combined with the decreasing area of ADM, it appears short-term practice of the texting task does alter the representation size and recruitment of corticospinal projections to the two small hand muscles in this study.

The conception of this study was based on the fact that cellular phone texting is such a prevalent means of communication in the current society. In the present iteration of cell phone design, the thumbs are solely used as the digits that interact with the virtual keyboard on touch screens. We hypothesized that this would result in enlarged cortical representations for the thumb, as seen in the FDI of proficient braille readers [11]. However, one confounding factor we did not anticipate is that all of our subjects were proficient texters (range 201–456 characters in two-minute FTT). An average keyboard typing speed on a computer is ~190 characters per minute (livechatinc.com), which shows our subjects were nearly as proficient on the cell phone as the average population is on a computer. With such a homogenous group, it could be expected that all of our subjects already had enlarged cortical representations and greater recruitment of corticospinal projections for the thumb. Identifying a control group in this study was not realistic for this subject population, as nearly everyone text messages on their cell phone. Several years prior, we had collected preliminary data for this study [19], and with only the 5 years that had elapsed, the subject population studied became significantly faster and more accurate at the FTT (See Table 1). Not only had the group gotten much better, the range of scores was much wider, suggesting that we missed the time when we could have seen associations between texting scores and our cortical measures. In addition, the fact that FTT scores were inversely correlated with age, a wider age range or older population may show less of a representation change and strength of projections for APB. An alternative explanation is that because all of our subjects were proficient in the keyboard task (FKT), a dexterous task using all the digits, the representations for all distal limb muscles are enlarged [20,21]. This would obscure any increases (APB) or decreases (ADM) we would have observed at the pre-test.

To achieve high-performance in a given skill, it is suggested that the cortical maps and underlying excitability (via corticospinal and intracortical connections) have to already be supported [9,20,22,23]. Given the proficiency of our subject population for texting, it is expected that the neural substrates in the brain for those specific thumb movements are already present and contributing to increased motor learning abilities [24]. This could also be an explanation for the short-term improvement observed in the FTT after only 10 min of practice. After practice, the cortical volume decreased, but the area of activity increased, showing a spread of activity throughout the digit representation of M1. Furthermore, the expansion of the area was correlated with the texting improvement, suggesting that the enlargement of the map drives the task improvement. There is evidence that the mechanisms underlying this spread of activity are from strengthening horizontal connections in M1 [3] from suppressing intracortical inhibitory circuits [6] or activating existing subthreshold excitatory connections [2]. However, the MEP size at 120% RTh also increased showing that the excitability of the corticospinal projections for the active cortical neurons increased. As this measure is not limited to just the excitability of the cortical neurons, but also the spinal motor neurons they are projected to, it is difficult to ascribe the short-term effects to just the cortex. Regardless, the mechanisms must be in place to allow the increase in texting performance.

One of the secondary hypotheses of this study was that the representation of ADM would be decreased in those individuals that were proficient at texting due to devoting more intracortical networks towards the thumb muscle and away from a control muscle on the opposite side of the hand [11]. The limiting factor between our study and that of the Braille readers by Pascual-Leone et al. was that our subjects were also very good at typing on a computer keyboard—a task that involves a high-level of sensorimotor control of the ADM muscle, along with other muscles controlling the digits. However, when the FTT was practiced for 10 min and the ADM was presumably not needed, the cortical measures of ADM decreased. It is unclear if we practiced the keyboard-typing task, instead of the texting task, if the decreased excitability and representations in ADM would have been prevented, or if they even would have shown the opposite change. This finding, however, coupled with the changes observed in APB after practice, do show the short-term reorganization that can occur in neighboring cortical networks for the improvement of a specific task.

There is also the issue of the accuracy of the skill being performed, as this influences the cortical representations more than simply increased use alone [4,25,26]. In the design of this study, this was addressed by turning off the ‘autocorrect’ feature on the cell phones. We expected more variable accuracy scores with the autocorrect off, but the combination of the very high accuracy (~98%) and the texting speed showed that the subjects really did learn the skill of texting. Even though all of our subjects had the autocorrect feature turned on when we got their cell phones, they did not seem to rely on it so much that it prevented the cortical representation changes and skill learning.

## 5. Conclusions

In conclusion, we presented a unique scenario where in healthy subjects the thumb is used excessively for a specific motor task, for a vast majority of the population. The use of the thumb in this way does change the motor cortex to enable the learning of the skill (text messaging). We do not know the long-term effects of this focused use of the thumbs, though it would not be surprising to start observing dystonia’s similar to those in musicians (involuntary thumb flexion) from the repetitive movements. This would be in addition to musculoskeletal issues such as tendinitis, myofascial pain syndrome and many others [27].

## Figures and Tables

**Table 1:** Comparison of pilot data collected from 2012 versus the data collected for this study (2017). None of the data was used twice. P-value listed at the bottom of the table.

Year	# of Subjects	FTT Score	Accuracy (%)
2012	N = 8	242.0 ± 115.8	93.1 ± 3.2
		(range 76—426)	(range 89.4—98.2)
			
2017	N = 18	346.6 ± 69.5	97.9 ± 1.5
		(range 201—456)	(range 94.9—100.0)
		*p* = 0.004	*p* < 0.001

## References

[1] Levine A.J., Lewallen K.A., Pfaff S.L. (2012). Spatial organization of cortical and spinal neurons controlling motor behavior. Curr. Opin. Neurobiol..

[2] Huntley G.W. (1997). Correlation between patterns of horizontal connectivity and the extend of short-term representational plasticity in rat motor cortex. Cereb. Cortex.

[3] Rioult-Pedotti M.-S., Friedman D., Hess G., Donoghue J.P. (1998). Strengthening of horizontal cortical connections following skill learning. Nat. Neurosci..

[4] Pascual-Leone A., Nguyet D., Cohen L.G., Brasil-Neto J.P., Cammarota A., Hallett M. (1995). Modulation of muscle responses evoked by transcranial magnetic stimulation during the acquisition of new fine motor skills. J. Neurophysiol..

[5] Muellbacher W., Ziemann U., Wissel J., Dang N., Kofler M., Facchini S., Boroojerdi B., Poewe W., Hallett M. (2002). Early consolidation in human primary motor cortex. Nat. Cell Biol..

[6] Rosenkranz K., Kacar A., Rothwell J.C. (2007). Differential modulation of motor cortical plasticity and excitability in early and late phases of human motor learning. J. Neurosci..

[7] Sanes J.N., Donoghue J.P. (2000). Plasticity and Prim. motor cortex. Annu. Rev. Neurosci..

[8] Classen J., Liepert J., Wise S.P., Hallett M., Cohen L.G. (1998). Rapid plasticity of human cortical movement representation induced by practice. J. Neurophysiol..

[9] Karni A., Meyer G., Rey-Hipolito C., Jezzard P., Adams M.M., Turner R., Ungerleider L.G. (1998). The acquisition of skilled motor performance: Fast and slow experience-driven changes in primary motor cortex. Proc. Natl. Acad. Sci. USA.

[10] Kleim J.A., Barbay S., Cooper N.R., Hogg T.M., Reidel C.N., Remple M.S., Nudo R.J. (2002). Motor learning-dependent synaptogenesis is localized to functionally reorganized motor cortex. Neurobiol. Learn. Mem..

[11] Pascual-Leone A., Cammarota A., Wassermann E.M., Brasil-Neto J.P., Cohen L.G., Hallett M. (1993). Modulation of motor cortical outputs to the reading hand of braille readers. Ann. Neurol..

[12] Ridding M., Rothwell J. (1997). Stimulus/response curves as a method of measuring motor cortical excitability in man. Electroencephalogr. Clin. Neurophysiol. Mot. Control..

[13] Oldfield R.C. (1971). The assessment and analysis of handedness: The Edinburgh inventory. Neuropsychologia.

[14] Wilson S., Thickbroom G., Mastaglia F. (1993). Transcranial magnetic stimulation mapping of the motor cortex in normal subjects. The representation of two intrinsic hand muscles. J. Neurol. Sci..

[15] van de Ruit M., Perenboom M.J., Grey M.J. (2015). TMS brain mapping in less than two minutes. Brain Stimul..

[16] Malcolm M., Triggs W., Light K., Shechtman O., Khandekar G., Gonzalezrothi L. (2006). Reliability of motor cortex transcranial magnetic stimulation in four muscle representations. Clin. Neurophysiol..

[17] Triggs W.J., Subramanium B., Rossi F. (1999). Hand preference and transcranial magnetic stimulation asymmetry of cortical motor representation. Brain Res..

[18] Wassermann E.M., McShane L.M., Hallett M., Cohen L.G. (1992). Noninvasive mapping of muscle representations in human motor cortex. lectroencephalogr. Clin. Neurophysiol. Potentials Sect..

[19] Riley Z.A., Hoseini N., Eckert N.R. (2013). Thenar cortical representation driven by cellular phone texting ability. Soc. Neurosci..

[20] Kleim J.A., Hogg T.M., Vandenberg P.M., Cooper N.R., Bruneau R., Remple M. (2004). Cortical synaptogenesis and motor map reorganization occur during late, but not early, phase of motor skill learning. J. Neurosci..

[21] Remple M.S., Bruneau R.M., Vandenberg P.M., Goertzen C., Kleim J.A. (2001). Sensitivity of cortical movement representations to motor experience: Evidence that skill learning but not strength training induces cortical reorganization. Behav. Brain Res..

[22] Tyč F., Boyadjian A., Devanne H. (2005). Motor cortex plasticity induced by extensive training revealed by transcranial magnetic stimulation in human. Eur. J. Neurosci..

[23] Nudo R., Jenkins W., Merzenich M., Prejean T., Grenda R. (1992). Neurophysiological correlates of hand preference in primary motor cortex of adult squirrel monkeys. J. Neurosci..

[24] Rosenkranz K., Williamon A., Rothwell J.C. (2007). Motorcortical excitability and synaptic plasticity is enhanced in professional musicians. J. Neurosci..

[25] Nudo R., Milliken G., Jenkins W., Merzenich M. (1996). Use-dependent alterations of movement representations in primary motor cortex of adult squirrel monkeys. J. Neurosci..

[26] Kleim J.A., Barbay S., Nudo R.J. (1998). Functional reorganization of the rat motor cortex following motor skill learning. J. Neurophysiol..

[27] Sharan D., Ajeesh P. (2012). Risk factors and clinical features of text message injuries. Work.

